# Exploring fundus‐controlled mesopic and scotopic perimetry in inherited retinal disease

**DOI:** 10.1111/aos.70142

**Published:** 2026-05-05

**Authors:** Laura J. Taylor, Amandeep S. Josan, Daniel A O Adeyoju, Isabella Farrance, Robert E. MacLaren

**Affiliations:** ^1^ Nuffield Laboratory of Ophthalmology, Department of Clinical Neurosciences University of Oxford Oxford UK; ^2^ Oxford Eye Hospital Oxford University Hospitals NHS Foundation Trust Oxford UK

**Keywords:** clinical trials outcome measures, microperimetry, retinitis pigmentosa, rod‐cone degeneration, two‐colour perimetry, visual fields

## Abstract

**Purpose:**

Microperimetry is increasingly used as an outcome measure in clinical trials for retinal disease. This study compares mesopic and scotopic microperimetry in a heterogeneous cohort of patients with inherited retinal disease to assess their suitability as clinical trial outcome measures and to determine the most appropriate testing modality.

**Methods:**

Participants completed mesopic and scotopic microperimetry (S‐MAIA) after 20 min of dark adaptation, as part of the Visual Function in Retinal Degeneration study (ISRCTN24016133). Testing was performed on both eyes (right first) without formal pupil dilation. Reliability and sensitivity performance were explored. A subset of participants (*n* = 23 patients and *n* = 16 controls) underwent repeat scotopic testing for repeatability analyses.

**Results:**

Twenty‐nine participants with inherited retinal disease and 40 healthy control participants completed microperimetry testing. Mesopic microperimetry in patients and in healthy controls showed good reliability and sensitivity performance. Scotopic microperimetry in patient participants was limited by poor test reliability, reflected by a high number of test exclusions from reliability screening, and significant floor effects in measured sensitivity. In addition, scotopic microperimetry showed no greater improvement in sensitivity or specificity than mesopic microperimetry. Repeatability analyses were limited by the small sample size following elimination of unreliable tests.

**Conclusion:**

Mesopic microperimetry is recommended as a stable and reliable outcome measure. Scotopic microperimetry appears to be limited by poor reliability and floor effects in patients with inherited retinal disease. The utility of scotopic microperimetry in patients with very early disease presentation, who present with highly preserved central vision (i.e. highly preserved mesopic microperimetry), remains unexplored.

## 1 INTRODUCTION

Inherited retinal diseases are the leading causes of visual impairment in the working population (Liew et al., 2014). Despite this, there is only one approved treatment for a very rare specific subtype, *RPE65‐associated* Leber Congenital Amaurosis (Deng et al., 2022). For most patients with inherited retinal diseases, no treatment is currently available, although clinical trials are ongoing. Many inherited retinal diseases present with rod‐cone degeneration; in these patients, visual acuity (VA) is preserved until late disease stages, rendering VA insensitive to early and subtle changes in visual function (Finger et al., 2019; Yang & Dunbar, 2021). Therefore, VA is broadly accepted to be unsuitable as a clinical trial outcome measure. Alternative visual function assessments are therefore required.

The ideal outcome measure is any test that can sensitively detect visual function changes as a result of therapeutic interventions, without being affected by natural patient variability and is acceptable to patients or participants (Coster, 2013). Microperimetry, also known as fundus‐controlled perimetry, provides an assessment of central macular sensitivity (Pfau et al., 2021). It has become a popular device for use as an outcome measure in clinical trials for inherited retinal disease, including *RPGR*‐associated RP (Yang & Dunbar, 2021). The Macular Integrity Assessment (MAIA) confocal microperimeter (CenterVue, Padova, Italy) combines a scanning laser ophthalmoscope, real‐time fundus tracking, and perimeter to accurately assess central retinal sensitivity. The posterior fundus is visualised via an infrared super‐luminescent diode, and fundus landmark features are registered and tracked in real time. Stimuli positions are altered dynamically to compensate for any fixational movements before presentation (Pfau et al., 2021).

Scotopic microperimetry is performed using an updated version of the standard MAIA microperimeter, the S‐MAIA (Scotopic Macular Integrity Assessment, Centervue S.p.A., Italy). It facilitates assessment of the central macular sensitivity at much lower light levels (background luminance <0.001 cd/m^2^) following a period of dark adaptation. Furthermore, it combines microperimetry testing with the concepts of two‐colour perimetry, presenting stimuli at two different wavelengths in turn (cyan: 505 nm followed by red: 627 nm), allowing for preferential isolation of rod versus cone dominant responses at individual retinal locations (Taylor et al., 2022).

To the best of our knowledge, this (and similar two‐colour static perimetry devices) are the only tests that provide subjective assessment of localised rod photoreceptor function. These are favourable over alternative global assessments of rod photoreceptor function, such as full‐field stimulus testing and rod testing in standard flash scotopic full‐field electroretinography, since more localised visual function testing is more likely to be sensitive to subtle changes due to disease progression or treatment‐related gains. Patients with retinal diseases, including inherited retinal disease, often report early symptoms of nyctalopia. Therefore, assessment of low‐light visual function via scotopic microperimetry may be a potentially useful tool and so warrants further exploration.

This study examines the suitability of mesopic microperimetry and scotopic microperimetry as outcome measures for clinical trials involving a mixed cohort of patients with rod‐cone degeneration resulting from inherited retinal disease. Participant test reliability is explored by evaluating fixation losses and fixation stability. In addition, the range of central retinal sensitivity is detailed using the standard sensitivity outputs and calculated volumetric hill of vision (Josan et al., 2021). Lastly, an assessment of mesopic and scotopic sensitivity and specificity to detect the disease versus the control cohort is performed. Overall, the suitability of microperimetry and scotopic microperimetry for use as an outcome measure will be evaluated, and recommendations made for future use.

## 2 MATERIALS AND METHODS

Participants were recruited as part of the Visual Function in Inherited Retinal Disease study (ISRCTN registration number: ISRCTN24016133, UK research ethics approval reference: 20/WM/0283) (Taylor, Josan, Stratton, et al., 2023). This study was conducted in accordance with the Declaration of Helsinki, and all participants provided informed written consent. The study focused on exploring outcome measures in patients with early mild‐to‐moderate rod‐cone generation secondary to inherited retinal disease. Patient participants with VA less than 6/60 or significant co‐pathologies were excluded. Healthy control participants included those with a VA of 6/7.5 or better and were excluded if they had any history of amblyopia or any previous ophthalmic disease or surgery with potentially lasting effects on visual function (as determined by a clinician). All examinations were carried out by a trained examiner.

### 2.1 Microperimetry

Mesopic microperimetry was completed first, using the S‐MAIA machine, in a darkened room (light level < 1.0 lux) without any formal dark adaptation or pupil dilation. Dark adaptation and pupil size have been shown not to have significant effects on measured central retinal sensitivity as long as the minimum pupil size of 2.5 mm is achieved (Han et al., 2017, 2019). A 1‐degree‐diameter red circle was used as the fixation target at the standard default fixed intensity. A standard 10–2 68‐point test grid was used with a 4–2 dB bracketing threshold strategy and Goldmann size III stimulus of various intensities (0–318 cd/m^2^) on a mesopic background (1.27 cd/m^2^). The stimulus dynamic range was 0–36 dB. Before examination, subjects were informed about the task and given verbal encouragement throughout testing. The non‐testing eye was occluded throughout. The right eye was tested first for each participant, followed by the left eye.

### 2.2 Scotopic microperimetry

Two‐colour scotopic microperimetry was performed with the same S‐MAIA device, this time using a 37‐point radial testing grid and a central 1‐degree fixation ring target. Unlike in mesopic testing, where the fixation target intensity was fixed, in scotopic testing, it was adjusted to ensure detection by the patient at the minimum possible level. Cyan stimuli testing was performed before red stimuli to minimise disrupting the dark‐adapted state of rods, and testing was performed with no pharmacological pupil dilation. The testing grid comprised three concentric rings displaced from a central point at 3, 5, and 7 degree radii. Stimuli were also presented using the 4–2 staircase bracketing strategy to obtain the final threshold sensitivity. The minimum and maximum luminance capability within the S‐MAIA for stimuli are 6.28 × 10^−5^ to 0.25 cd/m^2^, representing a dynamic range of 0–36 dB (Taylor et al., 2022). Before testing, subjects were also informed about the specific task and given verbal encouragement throughout testing. All participants underwent 20 minutes of dark adaptation prior to testing under scotopic conditions (<1.0 lux) (Montesano et al., 2021). The non‐testing eye was again occluded throughout, and the right eye was tested first for each participant, followed by the left eye.

### 2.3 Fixation stability and reliability indices

Both mesopic and scotopic microperimetry provide the same reliability metric outputs. Given the fundus tracking capability of the S‐MAIA, fixation location and stability (of the gaze) data are collected throughout testing at a refresh rate of 25 Hz. The fixation location is termed the preferred retinal locus (PRL); the MAIA measures this initially during the first 10 seconds of testing (denoted as PRL_i_, with i denoting initial and PRL_f_, with f denoting final). Large differences between PRL_i_ and PRL_f_ indicate unstable fixation (Morales et al., 2013).

Fixation stability outputs also include P1 and P2, which correspond to the percentage of fixation points falling inside 1° and 2° radii of the PRL, respectively (Morales et al., 2016). The MAIA standard output uses the P1 and P2 values to provide a fixation stability result based on the following classification: for fixation to be classified as stable, P1 must include >75% of fixation points. For a relatively unstable fixation, classification P1 must include <75% and P2 must include >75% of fixation points. An unstable fixation classification occurs when <75% of fixation points are within P2 (Fujii et al., 2002). The fixation stability classifications for each test were used in the reliability screening.

In addition, the bivariate contour ellipse area (BCEA) indicates the area and orientation of a two‐dimensional ellipse encompassing a given proportion (either 63% or 95%) of the fixation points. The smaller the BCEA value, the better the fixation stability. BCEA is the recommended metric for accurate fixation stability assessment (Crossland et al., 2009).

False positives were evaluated by fixation losses, recorded as the percentage of positive responses to a 10dB bright stimulus presented at the centre of the optic nerve head (Heijl‐Krakau method) (Heijl, 1987). Participants with fixation losses >30% were excluded from the final analysis.

Identification of the physiological rod‐free zone in the central fovea was used as an additional indicator of response reliability in this study (Taylor et al., 2024). Under scotopic conditions, it is assumed that cyan sensitivity at the central point should be significantly lower than any other pointwise sensitivity and would be expected to have 0.0 dB to be deemed a positive identification of the rod‐free zone. Lack of rod‐free zone identification suggests an additional false positive response, indicating poor reliability.

### 2.4 Repeatability assessment

A sub‐cohort of patients and controls underwent repeat scotopic microperimetry testing in the right eye only, using the same testing procedure as described above. The same examiner completed repeat testing to minimise inter‐operator bias. Patient participants completed repeat testing on the same day, generating data for intra‐session repeatability. However, control participants completed repeat testing within 4 weeks of the initial test, generating data for inter‐session repeatability. This deviation in repeat testing times between patients and controls was a result of practical limitations including participant availability and access to research equipment.

### 2.5 Statistical analyses

Non‐parametric analyses were used throughout, using both reliability data and sensitivity data. The standard output sensitivity indices are pointwise and mean sensitivity, measured in decibels (dB). Volume sensitivity, measured in decibel*degrees squared (dB*deg^2^), was calculated from the raw pointwise data for each patient examination, and using the freely available open‐source MAIA3D application (https://ocular.shinyapps.io/MAIA3D) for mesopic microperimetry and the equivalent application for scotopic microperimetry (https://ocular.shinyapps.io/scotopicMAIA/) (Josan et al., 2021). Analyses were completed in R (R: A Language and Environment for Statistical Computing, Vienna, Austria). Linear mixed model analysis using the lme4 package (Bates et al., 2015) and patient ID set as a random intercept variable (accounting for nested/repeated data) was used to compare the sensitivity results. To compare the sensitivity and specificity of each microperimetry test's ability to discriminate between patient and control cohorts, receiver operating characteristic (ROC) curves were generated using the pROC function and area under the curve (AUC) values were compared. Repeatability analyses were completed using standard Bland–Altman analyses for mean and volume sensitivity (Altman & Bland, 2017). A coefficient of repeatability (CoR) was identified for each index for each test. For pointwise repeatability, a linear mixed model framework was also used in conjunction with Bland–Altman analysis to again account for repeated measures (Josan, 2025).

## 3 RESULTS

Twenty‐two male and seven female patient participants with rod‐cone degeneration secondary to a genetically diagnosed inherited retinal disease (Table S1), median age 30 (IQR: 24–47) years, with median BCVA right eye 83 (IQR: 73–86) and left eye 79 (IQR: 71–84) ETDRS letters, completed mesopic and scotopic microperimetry testing. Three additional participants (four tests in total) failed to complete testing because they could not see the fixation target; the characteristics of these eyes are summarised in Table S2. Three of the four patient participant tests also had 0 dB mean microperimetry sensitivity, suggesting that they were at more advanced disease stages. A further four participants did not complete scotopic microperimetry testing on the left eye (tested second), one due to tiredness and three due to time restrictions.

Forty control participants, median age 24 years (IQR: 24–43), with median BCVA right eye 93 (IQR: 90–94) and left eye 92 (IQR: 89–94) ETDRS letters, completed mesopic and scotopic testing in the right eye and 39 in the left eye. One participant did not complete the left eye testing as their left eye was amblyopic and therefore ineligible. A further participant was recruited but was unable to complete any microperimetry tests due to their pupils being less than 2.5 mm in diameter, which is the minimum pupil diameter required by the device to obtain the retinal imaging to permit testing. Figure 1 summarises the number of tests completed.

**FIGURE 1:**
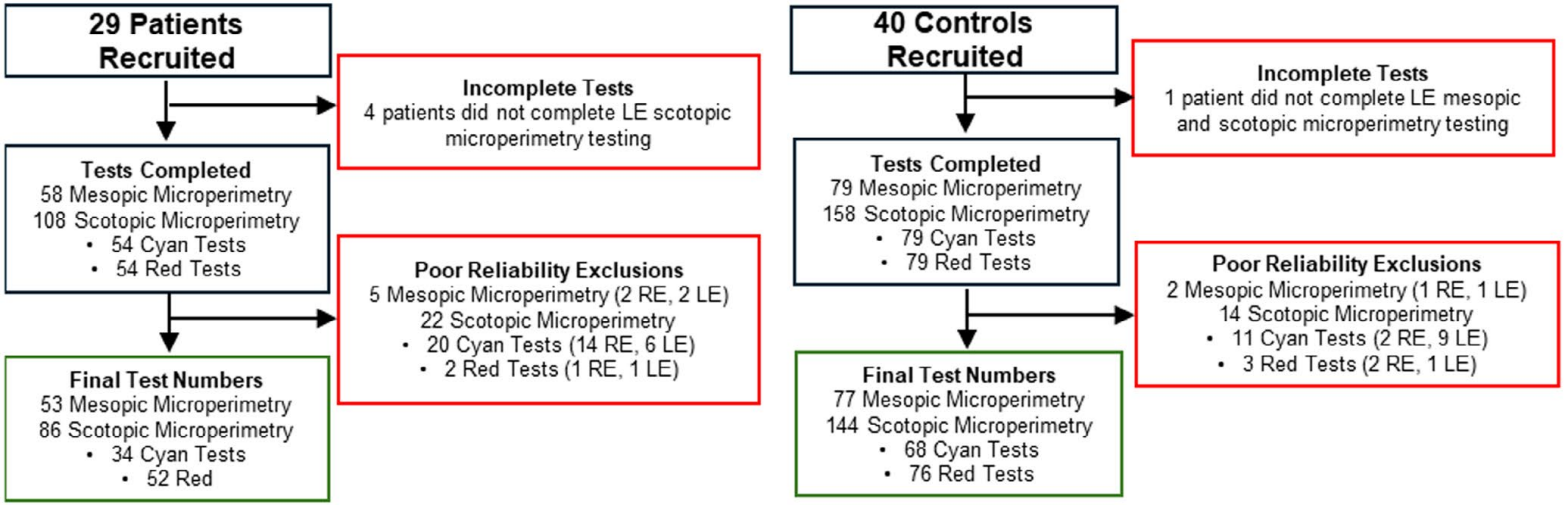
Details the number of participants, mesopic and scotopic microperimetry tests completed, excluded due to poor reliability and final test numbers.

A subset of 23 patient participants completed repeat scotopic microperimetry testing on the right eye only, generating a further 23 cyan tests and 22 red tests. Sixteen healthy control participants completed repeat cyan and red scotopic microperimetry on the right eye only.

## 4 RELIABILITY

Thirty‐seven per cent (20/54) of patient scotopic cyan tests and 3.7% (2/54) of patient scotopic red tests were excluded due to poor reliability (Figure 1). Of the excluded cyan tests, 70% were right eyes; since this is the first test performed, it could suggest a strong learning effect is present. However, this may be biased as four participants did not complete the left eye testing. Most participants were able to fixate steadily on the central 1‐degree red fixation target in both scotopic and mesopic microperimetry, as indicated by the low range of BCEA scores (Table S3). Only 7/22 (32%) of patient participant test exclusions resulted from unstable fixation (Table 1). False positives were the leading cause of test exclusion, arising from 12/22 (54%) exhibiting an unacceptable number of fixation losses, 9/22 (41%) participants showing no rod‐free zone detection and 4/22 (18%) demonstrating both unacceptable fixation losses and no rod‐free zone detection.

**TABLE 1:** Summary of reasons for excluded tests; some tests were excluded as they met more than one reason for exclusion.

	Patient participants			Healthy controls		
	Cyan	Red	Mesopic	Cyan	Red	Mesopic
Fixation losses > 30%	11	1	4	3	3	2
Unstable fixation	5	2	0	0	0	0
No rod‐free zone detected	10	NA	NA	10	NA	NA

Repeat tests	Patient participants		Healthy controls	
	Cyan	Red	Cyan	Red
Fixation losses > 30%	0	1	3	2
Unstable fixation	1	0	0	0
No rod‐free zone detected	3	NA	3	NA

Healthy controls showed more reliable performances, with 11/79 (14%) of scotopic cyan and 3/79 (4%) of scotopic red tests excluded (Figure 1). Six out of 14 (43%) were due to fixation losses, 10/14 (71%) due to no rod‐free zone detection and 2/14 (14%) due to both unacceptable fixation losses and no rod‐free zone detection. Interestingly, cyan left eye tests showed a high rate of exclusion due to no rod‐free zone detection. In scotopic testing, all participants showed stable fixation; lack of rod‐free zone detection was the leading cause of test exclusions (Table 1).

In comparison, mesopic microperimetry had considerably fewer test exclusions, with 8% (4/53) of mesopic microperimetry tests from patient participants being excluded due to high fixation losses, with one test being from the same participant with excluded scotopic tests due to no rod free zone detection. Similarly, only 2.5% (2/79) of healthy control mesopic microperimetry tests were excluded, both of which were due to high fixation losses only.

## 5 SENSITIVITY

All tests that were considered reliable were used in this sensitivity analysis (Table S4). This included those listed in Figure 1, as well as nine cyan patient tests and one control red test, who had an unreliable initial test but demonstrated a reliable repeat test.

All three tests show a negative inflated (zero‐inflated analogous) pointwise sensitivity distribution among patients, with unseen test loci making up 19.5% of mesopic testing, 41.7% of cyan stimuli, and 24.5% of red stimuli testing. Control participants showed a normal pointwise sensitivity distribution for both cyan and red, and none exhibited loci with −1.0 dB sensitivity (Figure 2).

**FIGURE 2:**
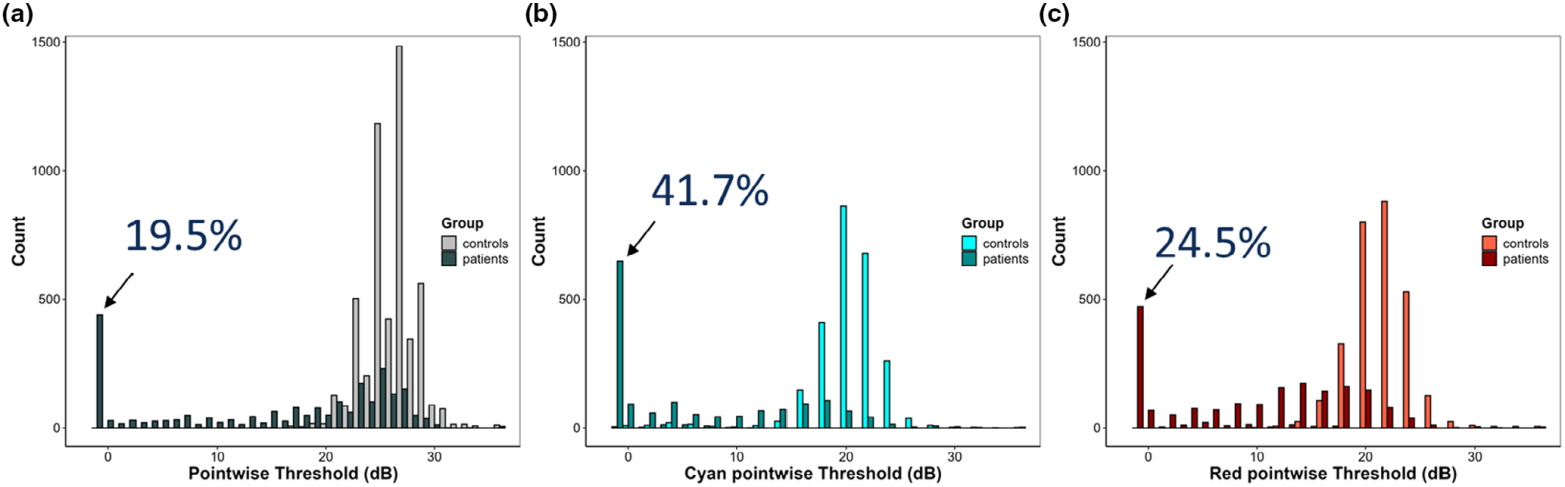
Frequency distribution plots for pointwise sensitivities reveal sub–zero‐inflated distributions in patients across mesopic (a), cyan (b) and red (c) tests, reflecting numerous loci with undetectable sensitivity, which are arbitrarily assigned as −1 dB by the MAIA device. In contrast, control participants exhibited normal distributions, with only a small number of low cyan sensitivities corresponding to the rod‐free zone. The percentages highlight the number of unseen test points (assigned −1.0 dB) from patient tests.

All participants (patients and healthy controls) had detectable mean and volume central sensitivity detectable with mesopic microperimetry. Nine patient participants had no detectable scotopic cyan mean sensitivity. Whereas only three patient participants showed no detectable scotopic cyan volume sensitivity, reflecting truly no detectable function within the device's capability. This highlights that six patients had detectable volume sensitivity despite no detectable mean sensitivity due to the averaging of numerous unseen points (assigned −1.0 dB), skewing the mean towards zero. All patient participants showed detectable red volume sensitivity, although mesopic microperimetry showed a much greater range of sensitivities (Figure 3). Volume sensitivity was significantly reduced in patients compared to controls for all tests (Linear Mixed Model: *p* < 0.0001) (Figure 3). In addition, all tests demonstrated high sensitivity and specificity in differentiating disease from control groups, with comparable AUC values (Figure 4). The optimum sensitivity threshold cut off (to classify individuals as ‘abnormal’) for mesopic microperimetry was 5632 dB.deg^2^ for 100% sensitivity and 96% specificity, for scotopic cyan the threshold cut off was 2542 dB.deg^2^ for 97% sensitivity and 90% specificity, and for red stimuli the threshold point was 2674 dB.deg^2^ for 100% sensitivity and 90% specificity. DeLong's test indicated no significant differences (*p* > 0.05) among the AUCs for each of the three tests, indicating all three tests are good and equal classifiers.

**FIGURE 3:**
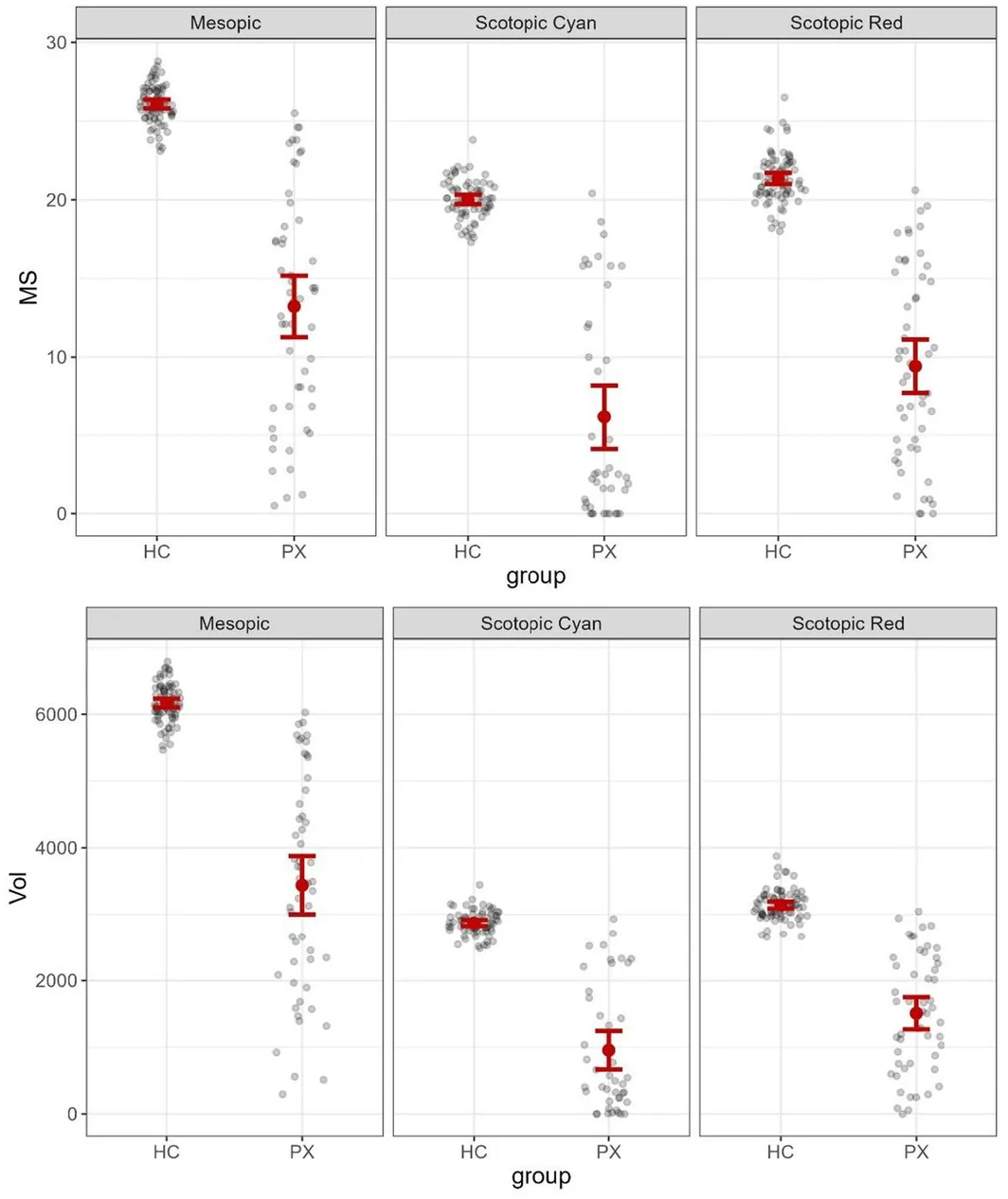
Distribution of mean sensitivity (MS) and volume sensitivity (Vol) values, with mean and 95% confidence intervals indicated by the error bars. HC, healthy control; PX, patient participant.

**FIGURE 4:**
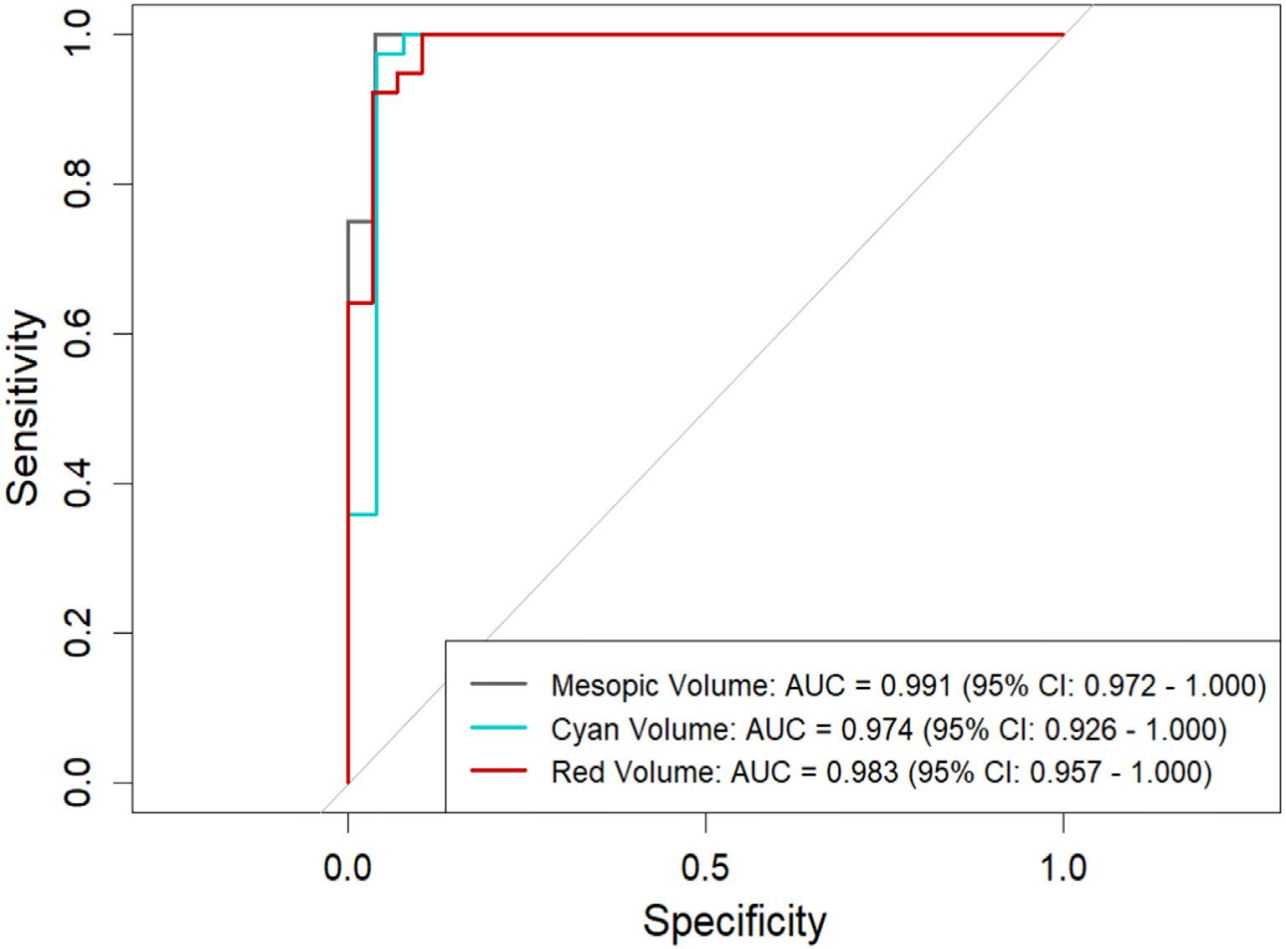
Receiver operator curve (ROC) analyses showing high sensitivity and specificity for mesopic, cyan and red scotopic microperimetry, using volume sensitivity.

**TABLE 2:** Patient CoR's for scotopic cyan and red sensitivity pointwise and volume indices.

	Patient inter‐session test retest variability (*n* = 10 cyan, *n* = 20 red)		Control intra‐session test retest variability (*n* = 11 cyan and red)	
	Bias (95% CI)	CoR (95% CI)	Bias (95% CI)	CoR (95% CI)
Pointwise (dB)				
Cyan	−1.2 (−2.4, 0.9)	12.4 (11.4, 13.2)	0 (−0.1, 0.3)	6.4 (5.8, 6.8)
Red	−0.4 (−0.2–0.2)	11.3 (10.6, 11.7)	0.1 (0.1, 0.5)	6.5 (5.9, 6.9)
Volume (dB.deg^2^)				
Cyan	175 (−300, 650)	1301 (895, 2376)	5 (−65, 75)	204 (142, 358)
Red	53 (−60, 167)	474 (361, 693)	−24 (−113, 64)	258 (180, 453)

## 6 REPEATABILITY

A subset of 23 patient participants completed repeat cyan and red testing on the right eye only for repeatability analyses. On repeat testing, reliability was substantially better, which could be indicative of a learning effect (Table S3). Only 5/45 (11%) tests were excluded, four cyan and one red, mainly due to high false positives. Sixteen healthy control participants completed repeat scotopic testing. Six repeat tests (out of 36) were excluded (four cyan and two red), again due to high false‐positive responses (Table 1). In total, only nine control participants and 10 patient participants had reliable Test 1 and Test 2 pairs that could be used in reliability analyses (Figures S1 and S2).

CoR was significantly higher in patients compared to controls, with both cyan and red stimuli (Table 2). Scotopic cyan had one patient who was a significant outlier. After excluding this outlier, the mean bias was −30.7 (95% CI: −139 to 78), the CoR for cyan volume reduced to 276 dB.deg^2^ (95% CI: 186–529), which is more comparable to control participant repeatability (Figure S3).

## 7 DISCUSSION

This study compared mesopic and scotopic microperimetry to assess whether scotopic microperimetry is a viable outcome measure in clinical trials for inherited retinal disease. Scotopic cyan testing exhibited a high exclusion rate due to poor test reliability, primarily driven by false positive responses, comprised of both fixation losses and lack of rod‐free zone detection. The high response to the novel rod‐free zone detection assessment suggests that the standard S‐MAIA reliability markers may not adequately detect poor reliability. Poor fixation stability did not appear to be a significant factor causing test exclusion. All participants showed detectable mesopic microperimetry, whereas more patient participants reached the device's floor effect scotopic cyan testing. Those unable to complete testing (including those with no detectable scotopic cyan sensitivity) tended to be at more moderate stages of vision loss, reflected by lower BCVA and mesopic microperimetry sensitivity (Table S2).

The higher exclusion rate for cyan compared to mesopic and scotopic red tests likely reflects the increased difficulty of performing the test. While repeat mesopic testing showed fewer exclusions, suggesting a learning effect, this may be influenced by selection bias, as only motivated patients opted for repeat testing. The inclusion of an additional rod‐free zone reliability marker for scotopic cyan also biased exclusion analyses, but highlights the potential value of incorporating enhanced false positive catch trials into mesopic and scotopic red testing. Montesano et al. (2021), proposed ‘wrong pressure events’ as an alternative reliability marker by analysing the raw fixation tracking data to identify responses outside a defined response window. This approach is comparable to the false positive assessments used in standard static automated perimetry (Olsson et al., 1997). While promising, it currently relies on post‐hoc computational analysis, which is of limited use in a clinical setting where the investigator needs to make an instant judgement on the test reliability. In addition, it is unclear how the ‘wrong pressure event algorithm’ allows for variation in testing and response times caused by delays triggered by the microperimeter eye tracking software. Nevertheless, incorporating such measures into standard outputs could improve false positive detection and test reliability.

Significant test–retest variability limits the utility of pointwise sensitivities. The practise of assigning −1.0 dB to non‐seen loci artificially lowers mean sensitivity, creating a false floor effect. This explains instances of 0.0 dB mean sensitivity despite measurable volume sensitivity, consistent with prior findings in patients with *RPGR*‐associated RP and choroideremia. These results support the use of volume sensitivity as a more robust index. This same limitation has been previously reported in a cohort of patients with *RPGR*‐associated RP undergoing MAIA mesopic microperimetry and S‐MAIA scotopic microperimetry in a cohort of patients with choroideremia, where they advocate for using volume sensitivity (Karuntu et al., 2025; Taylor et al., 2024; Taylor, Josan, Jolly, & MacLaren, 2023).

Although participants received thorough instructions, no formal learning test was performed. Mesopic microperimetry preceded scotopic testing, and good mesopic reliability was assumed to confer familiarity. However, high scotopic exclusion rates suggest that dark‐adapted testing remains challenging, requiring concentration to detect faint stimuli. Some improvements in left eye cyan performance and scotopic red stimuli may reflect learning effects, but the higher luminosity of red stimuli likely also contributes to better detectability. Previous studies have used a ‘training field under mesopic conditions’ or fast scotopic training exams before study testing and still experienced learning effects (Jolly et al., 2023; Montesano et al., 2021), which suggests insufficient training. Karuntu et al. (2025) showed greater mesopic microperimetry inter‐visit CoR in a mixed cohort of patients with retinitis pigmentosa, where the greater variability was attributed to greater time between visits (14 days), suggesting a loss of any learning effects. Further research is needed to determine whether a brief scotopic learning test, using threshold stimuli that mimic the actual test, can reduce learning effects. The duration of retained testing proficiency is unknown, raising the question of whether such a learning test should precede every session. It also remains unclear whether separate learning tests are necessary for both cyan and red stimuli.

On the other hand, learning effects do not explain the reduced scotopic cyan reliability seen in healthy control left eyes (tested second), which also showed a high number of false positives. Perhaps this is indicative of fatigue effects leading to greater false positive responses. The study did not collect repeatability data on mesopic microperimetry due to concerns about testing duration and the onset of fatigue effects. The test–retest variability of scotopic volume measurements for cyan and red stimuli, in both controls (inter‐visit) and patients (intra‐visit, after exclusion of a single cyan outlier), compares favourably with previously reported intra‐visit mesopic microperimetry repeatability, including a volume CoR of 324.2 dB.deg^2^ in patients with *RGRP*‐associated RP (Taylor, Josan, Jolly, & MacLaren, 2023) and an intra‐visit CoR of 255 dB.deg^2^ in a mixed RP cohort (Karuntu et al., 2025).

Scotopic microperimetry, using the S‐MAIA, is designed to compare cyan and red sensitivity, yet prior studies highlight substantial variability with this (Taylor et al., 2024). Additionally, in regions of complete rod dysfunction, low‐threshold cyan responses may arise from residual cones, as seen by non‐zero cyan threshold values measured in the rod‐free zone, complicating the interpretation of very low cyan sensitivities in patients with rod‐cone degeneration.

Repeat testing of patient participants was completed on the same day, whereas controls returned on separate days due to practical constraints. As a tertiary referral centre, many patients travel long distances to attend the Oxford Eye Hospital, making return visits challenging, whereas most controls were local students or staff for whom separate‐day testing was feasible. This discrepancy may limit the direct comparability of repeatability analyses between groups. The overall sample size was small, particularly for repeat testing after exclusion of unreliable tests. Recruitment was focused on patients with rod‐cone degeneration secondary to an inherited retinal disease, as opposed to specific inherited retinal disease genotypes. The range of genotypes (Table S1) was limited and a large number (12/29) of participants had choroideremia, which may affect the generalisability of the results. Pupil size was not formally controlled for. Prior work suggests dilation is unnecessary for mesopic microperimetry and scotopic testing that occurs under dark‐adapted conditions where pupils naturally dilate (Han et al., 2017). Despite this, one control participant was unable to complete testing due to small pupils (<2.5 mm). All participants completed mesopic microperimetry first, before completing 20 min of dark‐adaptation followed by scotopic microperimetry; this could have led to a confounding fatigue factor resulting in poor scotopic reliability. In hindsight, randomising the testing order would have eliminated this concern. Despite this, the improved reliability shown with repeat testing is more indicative of an underlying learning effect as opposed to fatigue effects.

Finally, this study did not evaluate the impact of variable fixation target intensity, which has been reported to influence the central cyan CoR and could affect sensitivity measurements under scotopic conditions (Pfau et al., 2017).

## 8 CONCLUSION

Mesopic microperimetry showed superior reliability and performance in both patients and healthy controls and is therefore recommended as an outcome measure for inherited retinal disease clinical trials. In patients with early mild‐to‐moderate visual impairment due to inherited retinal disease, scotopic microperimetry is limited by poor test reliability and significant floor effects. Scotopic microperimetry performance in healthy controls was better. Therefore, further studies are needed to evaluate scotopic microperimetry in patients with very early disease, who have well‐preserved central vision and preserved mesopic microperimetry.

## FUNDING INFORMATION

This project was funded by the National Institute for Health and Care Research (NIHR) under its (RfPB) Programme (Grant Reference Number NIHR202821) and the NIHR Oxford Biomedical Research Centre. The views expressed are those of the author(s) and not necessarily those of the NIHR or the Department of Health and Social Care.

## Supporting information


Table S1:
