# Supplementary material for: Exploring fundus‐controlled mesopic and scotopic perimetry in inherited retinal disease
Source: Acta Ophthalmol. 2026 May 5;104(7):e832–40. doi: 10.1111/aos.70142 (PMC13651910; doi:10.1111/aos.70142)
Supplement: Supplementary file 1 [file AOS-104-e832-s001.docx]

Supplementary

*Table S1: Range of genotypes causing inherited retinal disease in patient participant cohort.*

| **Genotype** | **n = 29** |
| --- | --- |
| **Retinitis Pigmentosa** |  |
| EYS | 1 |
| Rho | 1 |
| RPGR | 5 |
| USH2A | 4 |
| SNRNP200 | 1 |
| PRPF8 | 1 |
| CDHR1 | 2 |
|  |  |
| **Other Rod/Cone Degeneration** |  |
| CHM | 12 |
| Carrier LAMP2 | 1 |
| Carrier CHM | 1 |
|  |  |

*Table S2: Summary of participants unable to complete scotopic microperimetry testing*

| **Summary of participants unable to see the fixation target** | | | | |
| --- | --- | --- | --- | --- |
| **ID** | **Eye** | **VA (ETDRS letters)** | **Mesopic Microperimetry MS (dB)** | **Genotype** |
| 110 | RE | 32 | 1.6 | CHM |
| 118 | LE | 50 | 0.0 | RPGR |
| 139 | RE | 68 | 0.0 | USH2A |
| 139 | LE | 60 | 0.0 | USH2A |
|  |  |  |  |  |
| **Summary of patient participants scoring 0dB.deg^2^ Cyan Sensitivity** | | | | |
| 132 | RE | 82 | 12.1 | RPGR |
| 134 | RE | 70 | 1.0 | USH2A |
| 134 | LE | 70 | 1.2 | USH2A |

*Table S3, Summary of statistics for reliability markers for patient and participant scotopic and mesopic tests*

*Right eye (RE); Left eye (LE), Fixation losses (FL), Bivariate contour ellipse area (BCVA); Interquartile range (IQR)*

*Table S4 Number of tests included in sensitivity analysis*

|  | **mesopic tests (n)** | **cyan tests (n)** | **red tests (n)** |
| --- | --- | --- | --- |
| **Patients** | 53 | 43 | 52 |
| **Controls** | 77 | 69 | 77 |

*Figure S1: Bland Altman pointwise repeatability plots for patients (A and B) and controls (C and D) for cyan and red scotopic testing, respectively.*

*Figure S2: Bland Altman volume sensitivity repeatability plots for patients (A and C) and controls (B and D) for cyan and red scotopic testing, respectively.*

*Figure S3: Bland Altman plot for cyan volume sensitivity after single outlier removed.*
